# Enhanced cell migration and apoptosis resistance may underlie the association between high SERPINE1 expression and poor outcome in head and neck carcinoma patients

**DOI:** 10.18632/oncotarget.5032

**Published:** 2015-08-24

**Authors:** Miguel Angel Pavón, Irene Arroyo-Solera, Marta Téllez-Gabriel, Xavier León, David Virós, Montserrat López, Alberto Gallardo, Maria Virtudes Céspedes, Isolda Casanova, Antonio López-Pousa, Maria Antonia Mangues, Miquel Quer, Agustí Barnadas, Ramón Mangues

**Affiliations:** ^1^ Grup d'Oncogènesi i Antitumorals, lnstitut d'Investigacions Biomèdiques Sant Pau (IIB-Sant Pau), Barcelona, Spain; ^2^ Centro de Investigación Biomédica en Red en Bioingeniería, Biomateriales y Nanomecidicina (CIBER-BBN), Barcelona, Spain; ^3^ Department of Otorrinolaryngology, Hospital de la Santa Creu i Sant Pau, Barcelona, Spain; ^4^ Department of Otorrinolaryngology, Hospital Moises Broggi, Sant Joan Despí, Spain; ^5^ Department of Pathology, Clínica Girona, Girona, Spain; ^6^ Department of Medical Oncology, Hospital de la Santa Creu i Sant Pau, Barcelona, Spain; ^7^ Department of Pharmacy, Hospital de la Santa Creu i Sant Pau, Barcelona, Spain

**Keywords:** HNSCC, SERPINE1, prognosis, biomarker, AKT

## Abstract

High SERPINE1 expression is a common event in head and neck squamous cell carcinoma (HNSCC); however, whether it plays a role in determining clinical outcome remains still unknown. We studied SERPINE1 as a prognostic marker in two HNSCC patient cohorts. In a retrospective study (*n* = 80), high expression of SERPINE1 was associated with poor progression-free (*p* = 0.022) and cancer-specific (*p* = 0.040) survival. In a prospective study (*n* = 190), high SERPINE1 expression was associated with poor local recurrence-free (*p* = 0.022), progression-free (*p* = 0.002) and cancer-specific (*p* = 0.006) survival. SERPINE1 expression was identified as an independent risk factor for progression-free survival in patients treated with chemo-radiotherapy or radiotherapy (*p* = 0.043). In both patient cohorts, high SERPINE1 expression increased the risk of metastasis spread (*p* = 0.045; *p* = 0.029). The association between SERPINE1 expression and survival was confirmed using the HNSCC cohort included in The Cancer Genome Atlas project (*n* = 507). Once again, patients showing high expression had a poorer survival (*p* < 0.001). SERPINE1 over-expression in HNSCC cells reduced cell proliferation and enhanced migration. It also protected cells from cisplatin-induced apoptosis, which was accompanied by PI3K/AKT pathway activation. Downregulation of SERPINE1 expression had the opposite effect.

We propose SERPINE1 expression as a prognostic marker that could be used to stratify HNSCC patients according to their risk of recurrence.

## INTRODUCTION

Head and neck squamous cell carcinoma (HNSCC) is the sixth leading cancer in incidence worldwide [1, 2]. New treatment strategies that combine surgery, radiation and chemotherapy have improved organ preservation and patient quality-of-life [3]. However, 5-year survival has not markedly changed in the last two decades due to the high rate of loco-regional relapse and the development of metastasis or secondary tumors [1, 4]. Classical clinicopathological features are insufficient to predict clinical outcome or to identify patients that will benefit from standard treatment regimens. Therefore, the development of new predictive biomarkers could help to classify this heterogeneous group of tumors and improve treatment decision-making [5].

Extracellular matrix (ECM) remodeling is a frequent event during neoplastic transformation of epithelial cells and it is also associated with tumor malignancy, cell migration and invasion [6, 7]. The plasminogen activator (PA) system plays a central role in this process, in particular by regulating ECM proteolysis and degradation [8]. SERPINE1 (PAI-1) is the main regulator of the PA system, and it is also involved in signal transduction, tumor growth, invasion and metastasis [9]. It is the main inhibitor of the plasminogen activators tPA and uPA. Plasminogen activators (PA) stimulate the production of plasmin that in turn activates the fibrinolytic pathway and extracellular matrix degradation, leading to enhanced tumor cell migration [10, 11]. In several tumor types, SERPINE1 expression is up-regulated and it has been described as a poor prognostic marker [9, 12]. Besides its prognostic value, SERPINE1 expression has been validated as a marker for therapy decision making in patients with node-negative breast cancer [13, 14].

Previous studies pointed out that SERPINE1 expression increases during malignant transformation of squamous mucosa [15–19]. Gene expression profiles in HNSCC show that SERPINE1 is commonly over-expressed in primary tumors and lymph node metastasis [20–25]. However, the prognostic value of SERPINE1 in patients with HNSCC is still unknown. Although some studies have suggested an association between high SERPINE1 expression and poor prognosis [16, 20, 26–28], other authors have not found evidences of such an association [15, 18, 29, 30]. Inconclusive data reported to date could be related to small sample sizes or short follow-up data, and differences in patient characteristics or in the endpoint used to measure clinical outcome. Larger studies in patients with an accurate and longer clinical follow-up are therefore still necessary to establish the prognostic value of SERPINE1 in HNSCC.

On this basis, we studied the prognostic value of SERPINE1 expression, analyzing a retrospective (*n* = 80) and a prospective (*n* = 190) cohorts of HNSCC patients. We analyzed SERPINE1 expression in a third patient cohort obtained from The Cancer Genome Atlas database (*n* = 507). We also analyzed the effect of SERPINE1 expression on proliferation, migration and apoptosis induction in HNSCC cell lines.

## RESULTS

### High SERPINE1 protein expression is associated with a higher rate of metastasis development and poor clinical outcome

A total of 80 paraffin-embedded pre-treatment tumor biopsies, obtained from locally advanced patients with 68 months of median follow-up, were included in the retrospective immunohistochemical analysis (Table 1). Tumor cells showed membrane and cytoplasmatic positivity for SERPINE1 (Supplementary files, Figure S1). Tumor-adjacent normal tissue and stromal tissue areas were negative or showed negligible staining (Supplementary files, Figure S1).

**Table 1:** Characteristics of patients included in the retrospective study

Variable	All patients (*n* = 80)	High SERPINE1 (+++) (*n* = 29)	Intermediate or Low SERPINE1 (−/+/++) (*n* = 51)	*P* value^1^
**Sex**				
Men	76	26	50	0.182
Women	4	3	1	
**Age (years)**				
<60	30	11	19	0.952
>60	50	18	32	
**Tumor site**				
Oral cavity	7	5	2	0.095
Oropharynx	13	2	11	
Hypopharynx	13	4	9	
Larynx	47	18	29	
**Tumor size (T)**				
T2	8	4	4	0.690
T3	51	18	33	
T4	21	7	14	
**Node (N)**				
Positive	44	19	25	0.157
Negative	36	10	26	
**Tumor differentiation**				
Well	6	2	4	0.438
Moderate	67	26	41	
Poor	7	1	6	
**Tobacco**				
Non-smoker	4	2	2	0.560
<20 cigarette/day	5	1	4	
>20 cigarette/day	70	26	44	
Cigar or pipe	1	—	1	
**Alcohol**				
Non-drinker	13	7	6	0.152
<100 gr./day	32	10	22	
>100 gr./day	35	12	23	
**Local recurrence**				
Yes	15	8	7	0.111
No	65	21	44	
**Metastatic recurrence**				
Yes	9	6	3	**0.045**
No	71	23	48	
**Treatment**				
Radiotherapy	42	16	26	0.720
Surgery+/−RT	38	13	25	

1 Mann Whitney/Kruskal Wallis

**Figure 1:**
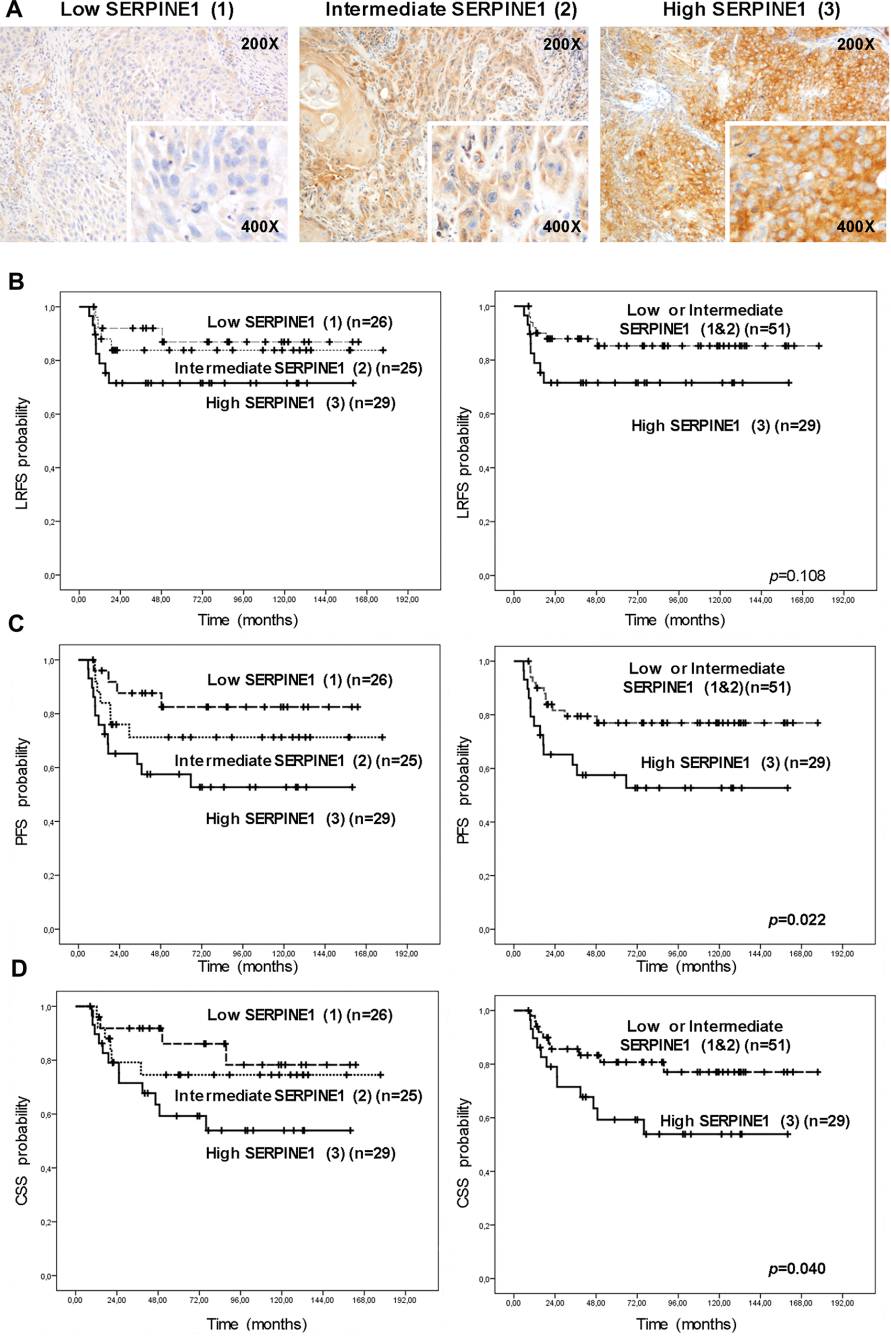
High protein SERPINE1 expression is associated with poor prognosis in patients with head and neck carcinoma included in a retrospective study **A.** Representative images of SERPINE1 immunohistochemistry in pre-treatment tumor biopsies included in the retrospective study (low intensity, 1; intermediate intensity, 2; high intensity, 3). Differences in local recurrence-free (LRFS) **B.** progression-free (PFS) **C.** and cancer-specific (CSS) survival **D.** according to the intensity of SERPINE1 staining.

Twenty-nine biopsies showed high SERPINE1 immunostaining intensity (3), 25 showed intermediate intensity (2), and 26 displayed low or negative staining (1) (Figure 1A). The percentage of SERPINE1 positive cells was similar in all samples (80–95%).

We observed a significant association between SERPINE1 immunostaining intensity and metastatic recurrences after treatment (*p* = 0.045) (Table 1). The rate of metastatic recurrences after treatment in patients with high SERPINE1 staining was higher than in patients with moderate or low staining.

SERPINE1 staining intensity was significantly associated with progression-free survival (PFS) (Figure 1C) and cancer-specific survival (CSS) (Figure 1D). Patients bearing tumors with high SERPINE1 staining intensity (3) had a shorter progression-free (PFS) (*p* = 0.022) and cancer-specific survival (CSS) (*p* = 0.040) than patients with tumors showing intermediate (2) or low (1) staining. There was a trend towards association between SERPINE1 staining intensity and local recurrence-free survival (LRFS), but this did not reach significance (*p* = 0.108) (Figure 1B). Only one oropharyngeal tumor was HPV positive in this patient cohort and was classified in the high SERPINE1 expression group. After performing an analysis that excluded this case we found that patients with high SERPINE1 expression continue having a significantly shorter progression-free survival than low expressing patients (*p* = 0.015) (Supplementary files, Figure S2).

### High SERPINE1 mRNA expression increases the risk of metastases development and is associated with poor outcome

Following the positive association found in the retrospective IHC study, we analyzed SERPINE1 mRNA expression in 190 tumor biopsies obtained from an independent cohort of HNSCC patients with 37 months of median follow-up (Table 2). We also analyzed SERPINE1 expression in 24 normal mucosa samples obtained from areas without visible lesions.

**Table 2:** Characteristics of patients included in the prospective study

Variable	All patients (*n* = 190)	High SERPINE1 (*n* = 114)	Low SERPINE1 (*n* = 76)	*P* value^1^
**Sex**				
Men	172	105	67	0.363
Women	18	9	9	
**Age (years)**				
<60	86	53	33	0.677
>60	104	61	43	
**Tumor site**				
Oral cavity	31	17	14	0.391
Oropharynx	65	43	22	
Hypopharynx	22	15	7	
Larynx	62	39	33	
**Tumor size (T)**				
T1	12	4	8	0.145
T2	60	34	26	
T3	73	45	28	
T4	45	31	14	
**Node (n)**				
Positive	111	73	38	0.054
Negative	79	41	38	
**Tumor differentiation**				
Well	13	5	8	0.085
Moderate	163	99	64	
Poor	14	10	4	
**Tobacco**				
Non-smoker	14	7	7	0.687
<20 cigarette/day	28	15	13	
>20 cigarette/day	146	91	55	
Cigar or pipe	2	1	1	
**Alcohol**				
Non-drinker	34	16	18	**0.037**
<100 gr./day	81	45	36	
>100 gr./day	75	53	22	
**Local recurrence**				
Yes	49	36	13	**0.028**
No	141	78	63	
**Metastatic recurrence**				
Yes	65	46	19	**0.029**
No	125	68	57	
**Treatment**				
Radiotherapy	51	27	24	0.124
CDDP-based CRT	74	52	22	
Cetux-based CRT	6	4	2	
Surgery+/−RT	59	31	28	

1 Mann Whitney/Kruskal Wallis

**Figure 2:**
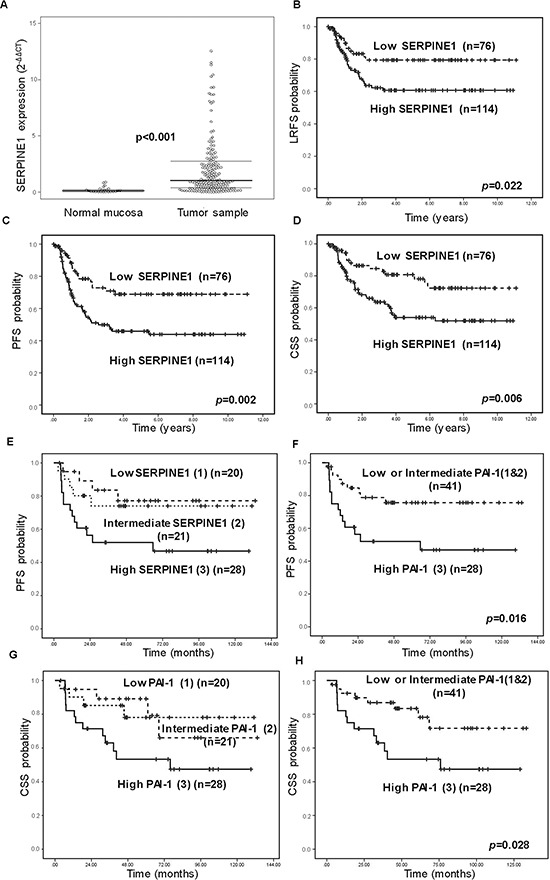
High SERPINE1 expression is associated with poor outcome in patients with head and neck carcinoma in a prospective study **A.** Differences in SERPINE1 mRNA expression between normal mucosa and the evaluated tumor samples. Differences in local recurrence-free (LRFS) **B.** progression-free (PFS) **C.** and cancer-specific survival (CSS) **D.** according to SERPINE1 mRNA expression (*n* = 190). Differences in progression-free (PFS) **E–F.** and cancer-specific (CSS) **G–H.** survival according to SERPINE1 immunostaining in 69 patients included in the prospective cohort.

SERPINE1 expression was significantly higher in tumor tissue than in normal mucosa samples (*p* < 0.001) (Figure 2A). Classification and regression-tree analysis method (CART) was used to establish the best cut-off to distinguish two groups of patients depending on SERPINE1 mRNA tumor levels and their probability of relapse (SERPINE1-mRNA level < or > 0.8). One hundred and fourteen patients had tumors with a SERPINE1 expression above the established threshold (high expression), whereas 76 patients had tumors with low SERPINE1 expression. The rate of metastatic recurrences was significantly higher in the group of patients with tumors expressing high levels of SERPINE1 (*p* = 0.029), thus confirming the results obtained in the IHC analysis (Table 2). Alcohol consumption (*p* = 0.036) and local recurrence (*p* = 0.028) were also associated with SERPINE1 expression.

Patients with high SERPINE1 tumor expression had shorter LRFS (*p* = 0.022), PFS (*p* = 0.002) and CSS (*p* = 0.006) than patients with low SERPINE1 expression (Figure 2). Multivariate Cox model analysis showed that SERPINE1 expression (HR 1.73, 95%CI 1.02–2.92, *p* = 0.042), tumor size (HR 2.18, 95%CI 1.29–3.70, *p* = 0.004) and node involvement (HR1.88, 95%CI 1.13–3.16, *p* = 0.016) were independent risk factors for progression-free survival (Table 3). Moreover, tumor size (HR 1.78, 95%CI 0.99–3.18, *p* = 0.050) and node involvement (HR 2.23, 95%CI 1.22–4.07, *p* = 0.009) were identified as independent risk factors for survival (Table 3). There was a clear trend towards significance in the association between high SERPINE1 expression and poor patient survival (HR 1.78, 95%CI 0.98–3.23, *p* = 0.057), however the differences observed among groups, did not reach statistical significance in the multivariate analysis. Multivariate Cox analysis, excluding patients treated with surgery, showed that SERPINE1 expression (HR 1.92, 95%CI 1.03–3.59, *p* = 0.043) and tumor size (HR 2.39, 95%CI 1.29–4.39, *p* = 0.005) were independent risk factors for progression-free survival in patients receiving radiotherapy and chemo-radiotherapy as the main treatment option (Table 3).

**Table 3:** Multivariate Cox model analysis in patients included in the prospective analysis (*n* = 190)

All Patients (*n* = 190)				
	Progression- free survival (PFS)		Cancer-specific survival (CSS)	
	HR (95% CI)	**p* value*	HR (95% CI)	**p* value*
**Sex**	1.02 (0.39–2.61)	0.976	1.76 (0.72–4.30)	0.217
**Tumor size (T)**	2.18 (1.29–3.70)	**0.004**	1.78 (0.99–3.18)	**0.050**
**Node (N)**	1.88 (1.13–3.16)	**0.016**	2.23 (1.22–4.07)	**0.009**
**Age**	0.86 (0.54–1.37)	0.514	0.767 (0.46–1.29)	0.320
**SERPINE1**	1.73 (1.02–2.92)	**0.042**	1.78 (0.98–3.23)	0.057

Radiotherapy/Chemoradiotherapy treated (*n* = 131)				
	Progression- free survival (PFS)		Cancer-specific survival (CSS)	
	HR (95% CI)	**p* value*	HR (95% CI)	**p* value*
**Sex**	1.13 (0.39–3.29)	0.976	1.76 (0.58–5.30)	0.317
**Tumor size (T)**	2.39 (1.29–4.39)	**0.005**	1.95 (0.97–3.89)	0.059
**Node (N)**	1.71 (0.94–3.11)	0.077	2.41 (1.15–5.06)	**0.020**
**Age**	0.83 (0.48–1.44)	0.507	0.84 (0.443–1.59)	0.594
**SERPINE1**	1.92 (1.03–3.59)	**0.043**	1.80 (0.86–3.78)	0.117

Sex = Male versus Female; Tumor size = T3-T4 versus T1-T2; Node (N) = positive node versus negative node; Age: < 60 years versus > 60 years; SERPINE1 = high versus low expression

HR = Hazard Ratio; 95% IC = 95% Confidence Interval;

HPV status was analyzed in patients with oropharyngeal cancer treated at Hospital de la Santa Creu i Sant Pau (HSCSP). Thirty-five tumors were HPV negative, 9 HPV positive and HPV status was not available in 20 patients. SERPINE1 maintained its value as a marker of progression-free survival, when we analyzed the mRNA data from the prospective RT-PCR patient cohort, after excluding patients with oropharyngeal HPV positive tumors or oropharyngeal tumors which HPV status was unknown (Supplementary files, Figure S2). Patients with high SERPINE1 expression had a significantly progression-free survival than patients with low SERPINE1 expression (*p* = 0.015).

We analyzed SERPINE1 expression in a subgroup (*n* = 69) of patients included in the prospective study by immunohistochemistry. Again, SERPINE1 staining intensity was significantly associated with progression-free survival (PFS) (*p* = 0.016) and cancer-specific survival (CSS) (*p* = 0.028), confirming the results obtained by RT-PCR (Figure 2E–2H).

### SERPINE1 expression was associated with poor survival in a third cohort of HNSCC patients included in The Cancer Genome Atlas database

**Figure 3:**
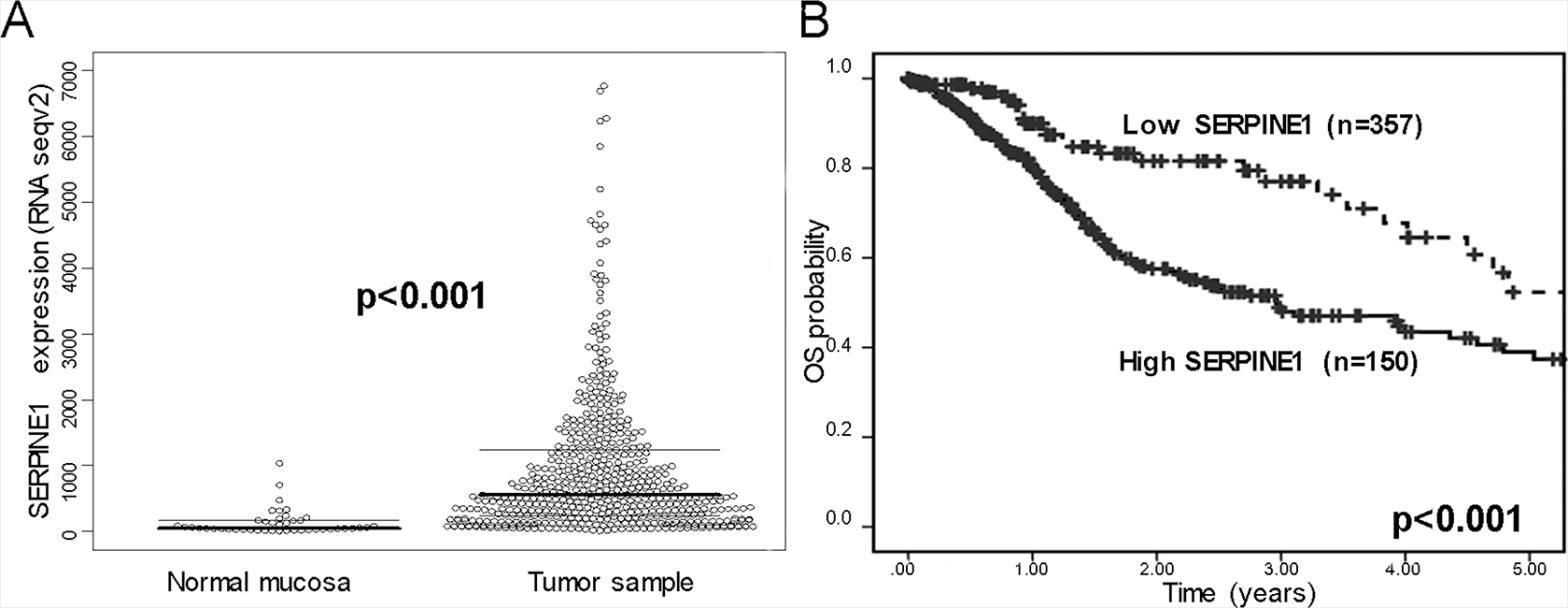
SERPINE1 expression in patients included in the Cancer Genome Atlas Database (TCGA) **A.** Differences in mRNA expression of SERPINE1 between normal mucosa (*n* = 44) and primary tumor samples (*n* = 520) of the HNSCC patients included in The Cancer Genome Atlas (TCGA) database. **B.** Differences in overall survival between patients, included in TCGA database, bearing tumors with low or high SERPINE1 tumor expression.

We analyzed SERPINE1 expression in an independent cohort of HNSCC patients having RNA sequencing results from 520 primary tumors and 44 mucosa samples recorded in The Cancer Genome Atlas (TCGA) database. SERPINE1 expression was significantly higher in tumor than in mucosa samples (*p* < 0.001) (Figure 3A). Kaplan -Meier curves showed that patients with high SERPINE1 tumor expression had shorter survival than patients with low SERPINE1 tumor expression (*p* < 0.001) (Figure 3B).

Univariate Cox model analysis showed that overall survival was significantly lower in patients whose tumors expressed high level of SERPINE1 than in patients with low SERPINE1 expression (HR:2.03, 95%CI 1.32–3.10, *p* = 0.01). Multivariate Cox analysis showed that SERPINE1 expression (HR:1.73, 95%CI 1.05–2.79, *p* = 0.027) and pathologic N classification (HR:1.58, 95%CI 1.09–2.31, *p* = 0.017) were identified as independent risk factors for death in this HNSCC patient cohort (Table 4). Other clinical variables such as sex, tumor size and age were not associated with patient survival (Table 4). Differences in survival between high and or low expressing tumors remained statistically significant in an analysis of the TCGA patient cohort that excluded HPV positive tumors (*n* = 20). Patients with tumors showing a high expression of SERPINE1 continue to have a significantly (*p* = 0.001) higher risk of death than patients with low expression (Supplementary files, Figure S2).

**Table 4:** Multivariate Cox model analysis for overall survival in patients included in TCGA database (*n* = 507)

	Cox Univariate		Cox Multivariate	
	HR (95% CI)	*p value*	HR (95% CI)	*p value*
**Sex**	1.23(0.88–1.71)	0.225	1.09 (0.73–1.64)	0.665
**Tumor size (T)**	1.22(0.87–1.71)	0.233	1.25 (0.81–1.92)	0.311
**Pathologic N**	1.62(1.11–2.35)	**0.012**	1.58 (1.09–2.31)	**0.017**
**Age**	1.30(0.94–1.81)	0.112	1.31 (0.89–1.93)	0.169
**SERPINE1**	2.03(1.32–3.10)	**0.010**	1.725 (1.06–2.79)	**0.027**

Sex = Male versus Female; Tumor size = T3-T4 versus T1-T2; Pathologic *N* = positive node versus negative node; Age: > 60 years versus < 60 years; SERPINE1 = high versus low expression

HR = Hazard Ratio; 95% IC = 95% Confidence Interval.

Interestingly, in the TCGA series, SERPINE1 expression was significantly associated with the presence of perineural invasion (PNI) (*p* < 0.001, Fisher test). PNI-positive rate was higher in tumors with high SERPINE1 expression (54%) than in tumors expressing low levels (23.9%). The rate of lymphovascular invasion-positive tumors was higher in tumors with a high SERPINE1 expression (37%) than in tumors with a low expression (28%), however, differences between groups for this variable did not reach statistical significance (*p* > 0.05).

### SERPINE1 expression inhibits cell proliferation and enhances migration in head and neck carcinoma cell lines

**Figure 4:**
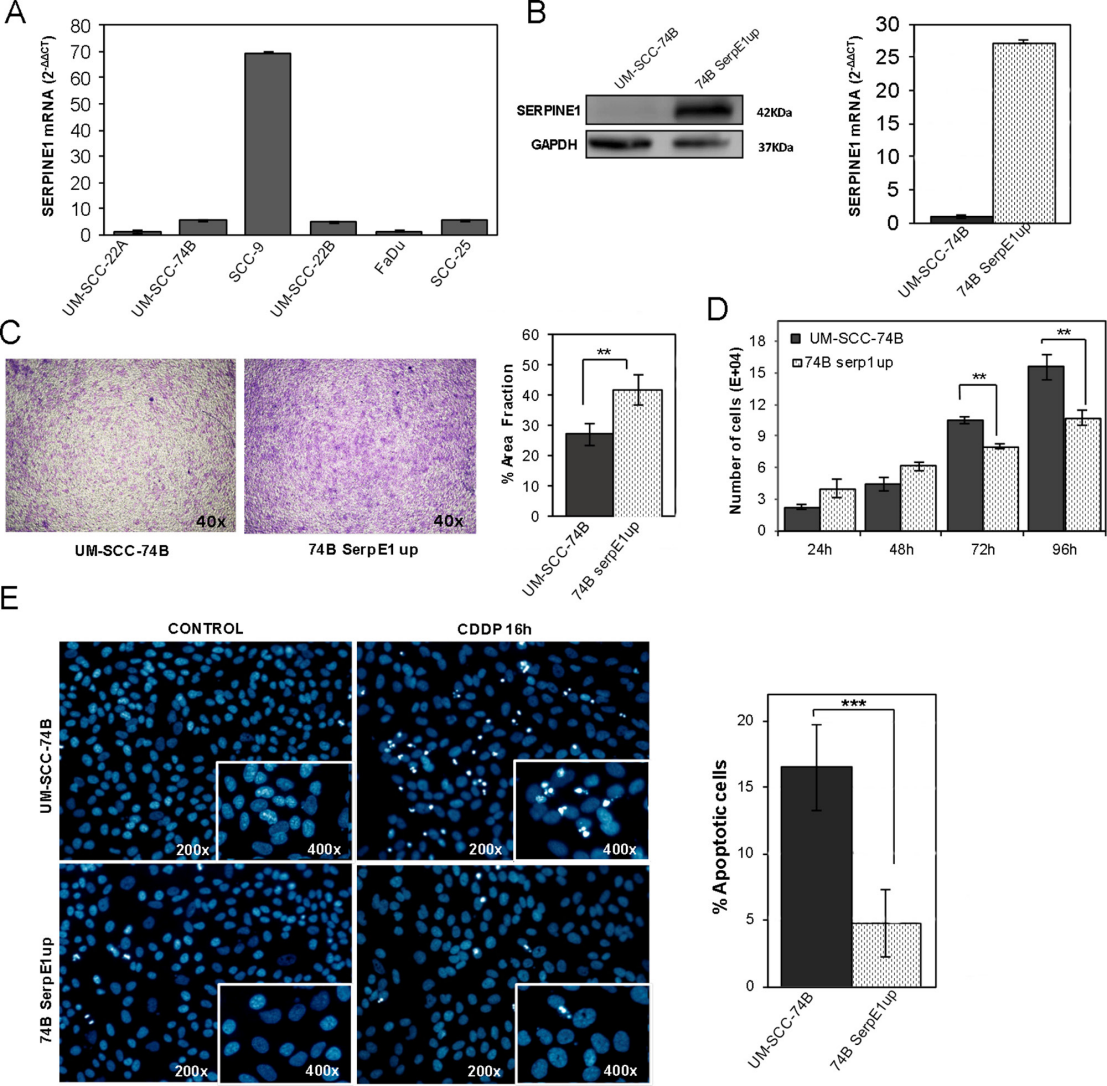
Ectopic over-expression of SERPINE1 increases migration, reduces proliferation and inhibits apoptotic induction in the UM-SCC-74B HNSCC cell line **A.** SERPINE1 mRNA levels in six HNSCC cell lines. **B.** SERPINE1 over-expression in the stably transduced UM-SCC-74B cell line (74B-SerpE1up), as analyzed by Western-Blot (left) and RT-PCR (right). Transwell migration **C.** and cell proliferation **D.** assays in UM-SCC-74B and 74B-PAI1up cells. **E.** Representative images of DAPI stained nuclei in UM-SCC-74B and 74B-SerpE1up cells before and after 16 hours of 15 μM cisplatin treatment (left) Over-expression of SERPINE1 reduces the number of apoptotic figures in cisplatin-treated cells (right). **p < 0.01 and ***p < 0.001.

We analyzed SERPINE1 mRNA expression in six head and neck squamous cell carcinoma cell lines (UM-SCC-22A, UM-SCC-22B, UM-SCC-74B, FaDu, SCC9 and SCC25) (Figure 4A). The mean SERPINE1 mRNA level was 15.49 within a range of 1.00–69.24. The SCC9 cell line displayed the highest SERPINE1 expression whereas the rest of cell lines expressed similarly low SERPINE1 mRNA levels.

We next sought to determine whether the ectopic over-expression or inhibition of SERPINE1 could affect cell proliferation and migration. We generated a UM-SCC-74B cell line stably over-expressing SERPINE1 (74B-SerpE1up) by transducing it with the pFUGW_SERPINE1 lentiviral vector (Figure 4B). Transwell assays showed that SERPINE1 over-expression increased the migration capacity of the UM-SCC-74B cell line (*p* = 0.004) (Figure 4C). Moreover, cell proliferation was reduced in cells over-expressing SERPINE1 at 72 hours (*p* < 0.01) and 96 hours after seeding (*p* < 0.01) (Figure 4D).

**Figure 5:**
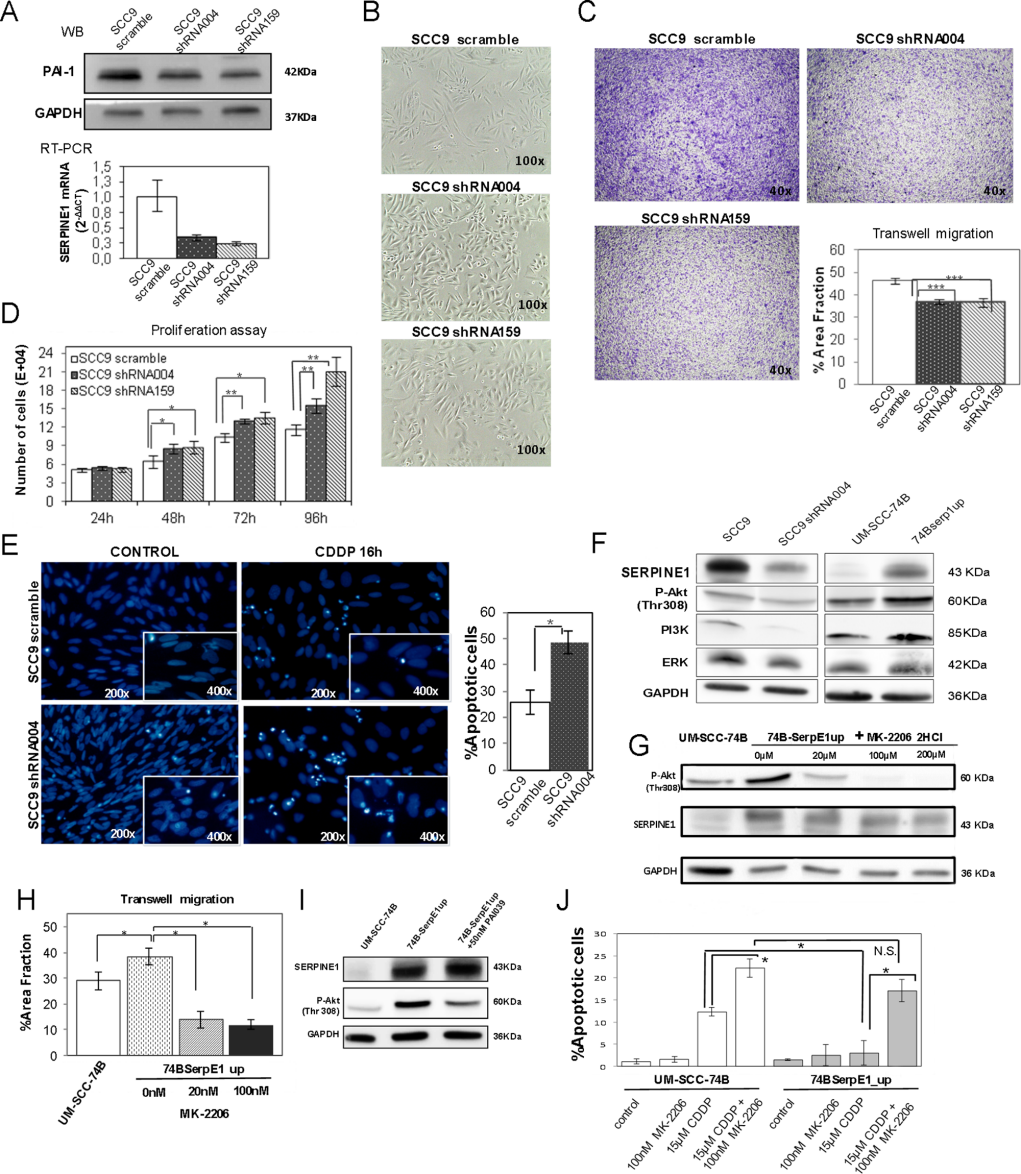
PAI-1 knockdown in the SCC9 cell line decreases migration, enhances proliferation and promotes apoptotic induction **A.** Expression of SERPINE1 in SCC9 and cells transduced with shRNAi (SCC9 shRNA004 and SCC9 shRNA159, as assessed by Western-Blot (above) or RT-PCR (bellow); **B.** Representative images showing changes in cell morphology after the inhibition of SERPINE1 expression; **C.** Transwell migration and **D.** proliferation assays in SCC9, SCC9 shRNA004 and SCC9 shRNA159 cells; **E.** Representative images of DAPI stained nuclei before and after 16 hours of 40 μM cisplatin treatment in SCC9 and shRNA transduced cells, showing higher apoptosis in PAI-1 downregulated cells; **F.** Western blot analysis of P-Akt, PI3K and ERK in SCC9, SCC9 shRNA004, UM-SCC-74B and 74BSerp1up cells; **G.** western blot analysis of P-Akt in cells treated during 48 hours with the AKT inhibitor MK-2206; **H.** Transwell migration assays in cells treated with the MK-2206 inhibitor; **I.** SERPINE1 and P-Akt protein expression in cells treated 48 hours with the SERPINE1 inhibitor PAI-039. **J.** Combination of cisplatin and AKT inhibitor MK2206 in UM-SCC-74B and 74BSerpE1up cells.*p < 0.05

The SCC9 cell line was selected for SERPINE1 down-regulation using lentiviral transduction with two shRNA constructs (TRC2_331004, TRC2_370159). SCC9shRNA004 and SCC9shRNA159 transduced cells that stably expressed shRNA showed inhibition of SERPINE1 expression (Figure 5A). The Inhibition of SERPINE1 expression analyzed by RT-PCR was 65% for the SCC9shRNA004 and 75% for the SCC9shRNA159 (Figure 5A).

After SERPINE1 inhibition, a partial loss of its characteristic fusiform shape was observed in the SCC9 cell line (Figure 5B). Inhibition of SERPINE1 expression by shRNA reduced migration in SCC9shRNA004 and SCC9shRNA159 transduced cells (*p* < 0.001 and *p* < 0.001, respectively) (Figure 5C). Cell proliferation was significantly higher in SCC9shRNA004 and SCC9shRNA159 cells than in the parental SCC9 cells at 48 hours, 72 hours and 96 hours after seeding (Figure 5D).

We analyzed the activation status of the PI3K/AKT cell signaling pathway in cells over-expressing or under-expressing SERPINE1. The 74B-SerpE1up cells, which over-expressed SERPINE1, showed higher levels of AKT phosphorylation than the parental UM-SCC-74B cell line, whereas SCC9shRNA004 and SCC9shRNA159 transduced cells, that under-expressed SERPINE1, had lower levels of AKT phosphorylation than the SCC9 scramble cell line (Figure 5F). The treatment of 74BSerpE1up cells during 48 hours with a specific AKT inhibitor (MK-2206) reduced AKT phosphorylation (Figure 5G) and reverted the increased migration observed in cells over-expressing SERPINE1 (Figure 5H). Moreover, after 48 h of exposure to 50 nM PAI-039, a specific inhibitor of SERPINE1, we observed a downregulation of p-AKT in 74BSerpE1up cells over-expressing SERPINE1 (Figure 5I).

### Ectopic over-expression of SERPINE1 protects cells from cisplatin–induced apoptosis

The 74B-SerpE1up cells, which over-expressed SERPINE1, were less sensitive to cisplatin treatment than UM-SCC-74B cells. After 48 hours of exposure to cisplatin, the IC50 increased from 10 μM to 20 μM by the ectopic expression of SERPINE1. We analyzed the number of apoptotic bodies (nuclear condensation) in UM-SCC-74B and 74B-SerpE1up cells after 16 hours of cisplatin treatment. Cells over-expressing SERPINE1 (74B-SerpE1up) showed fewer apoptotic bodies than the parental UM-SCC-74B cells (Figure 4E). In line with these results, the inhibition of SERPINE1 expression in SCC9 cells significantly increased cisplatin-induced apoptosis. The number of apoptotic bodies after cisplatin exposure in the SCC9shRNA004 and SCC9shRNA159 transduced cells was higher than in SCC9 scramble cells (Figure 5E). The number of apoptotic bodies observed after treatment with the combination of cisplatin (48 h exposure) with the AKT inhibitor MK2206 (72 h exposure), was significantly higher in the UM-SCC-74B or 74BSerpE1up cells than in the corresponding cells treated with cisplatin alone (48 h exposure) (Figure 5J). The differences between groups were more intense in cells over-expressing SERPINE1 (74B-SerpE1up) in which the combination of cisplatin with the AKT inhibitor MK2206 completely reverted their resistance to apoptosis.

The combination of cisplatin with the AKT inhibitor MK2206 completely reverts the resistance of 74BSerpE1 cells (overexpressing Serpine1) to cisplatin, since it induces apoptosis at a level similar to that achieved in UM-SCC-74B cells after their treatment with the same combination (Figure 5J).

## DISCUSSION

We identified SERPINE1 expression as a poor prognostic marker in head and neck squamous cell carcinoma. A high expression of SERPINE1 increased the risk of metastasis and was associated with a poor clinical outcome.

We obtained these results analyzing two independent patient cohorts with head and neck cancer (*n* = 80, *n* = 190). We used a third patient cohort of HNSCC (*n* = 507) included in TCGA database to support the association between SERPINE1 expression and patient survival. Despite using different methods to detect SERPINE1 expression in pre-treatment tumor biopsies, we observed a positive association between high SERPINE1 expression and poor clinical outcome in three independent patient cohorts. Taken together, our results show that SERPINE1 expression has a strong prognostic value in patients with head and neck carcinoma. We also identified SERPINE1 expression as an independent risk factor for tumor progression in patients treated with radiotherapy or chemo-radiotherapy.

We confirmed the prognostic value of SERPINE1 expression excluding HPV tumors from the survival analysis in the three patient cohorts. Therefore, the results of this study could be particularly relevant in HPV negative HNSCC population. Due to the low incidence of HPV positive tumors detected in our patient cohorts, which is a finding consistent with our geographical area [31], we could not assess whether SERPINE1 is or not a prognostic marker in HPV positive patients. Future studies in a large HNSCC patient cohort bearing HPV positive tumors will be needed to assess this possibility.

Evidence on the value of SERPINE1 expression as a prognostic factor in head and neck cancer has been inconclusive to date. In line with our results, Speleman and colleagues showed that high expression of SERPINE1 was associated with shorter disease-free survival in a univariate analysis performed in 46 HNSCC patients [16]. Magnussen and colleagues identified SERPINE1 and uPAR expression as predictive markers of disease specific death in early stage oral carcinomas (*n* = 26) [26]. However, due to the relatively small number of patients analyzed and the lack of a multivariate analysis, these two studies did not reveal whether SERPINE1 could be used as an independent marker to predict the risk of disease relapse. Three further studies also found that SERPINE1 expression was associated with HNSCC prognosis but only when it was analyzed together with additional markers, such as uPA, SPARC or SMA [20, 27, 28]. In contrast, many other studies found no association between SERPINE1 and clinical outcome in HNSCC patients [15, 18, 29, 30]. Our study may have helped towards resolving this controversy because it has identified SERPINE1 expression as a strong independent prognostic marker in patients with HNSCC.

Our results are consistent with those reported in other cancer types. SERPINE1 has been associated with poor clinical outcome in colon, breast, gastric, cervical, esophageal, lung, ovarian and thyroid cancers [9, 14, 32–37]. Similarly to our findings in HNSCC, a high expression of SERPINE1 was found to increase the risk of developing metastasis in patients with node negative breast cancer.

We have shown that the ectopic over-expression of SERPINE1 promotes cell migration, whereas the inhibition of SERPINE1 expression generates the opposite effect, reducing the migration capacity of HNSCC cell lines. The observed association between high SERPINE1 expression and enhanced cell migration may seem counterintuitive regarding the notion that PA inhibition could reduce ECM degradation and cell invasion. However, previous studies have shown that SERPINE1 promotes cell migration through its inhibitory activity against plasmin, preserving the stromal architecture and providing traction for cancer cells during migration [38]. Furthermore, SERPINE1 can also improve cell migration by a mechanism independent of the fibrinolytic pathway [39–41]. *In vitro* results, showing that SERPINE1 over-expression increases migration in head and neck tumor cells, are in agreement with the higher risk of metastatic recurrence observed in patients bearing tumors with a high expression of SERPINE1. They are also consistent with perineural invasion being more frequent in tumors over-expressing SERPINE1 in the TGCA cohort, which also support the role of this protein in head and neck tumor dissemination.

We also showed that cells over-expressing SERPINE1 were less sensitive to cisplatin treatment; one of the main drugs included in most chemotherapy protocols for the treatment of HNSCC patients. Interestingly, we observed an activation of the AKT pathway in cells over-expressing SERPINE1 that could be responsible for the stimulation of cell migration and the protection of HNSCC cells from cisplatin-induced apoptosis. Similar findings have been reported in breast carcinoma, human promyeolocityc leukemia, prostate carcinoma and fibrosarcoma cells. In these tumor types a high expression of SERPINE1 protects cells from chemotherapy-induced apoptosis and associates with the activation of the PI3K/AKT/mTOR signaling pathway [42–44]. Consistently, activation of this pathway is emerging as an important oncogenic mechanism in HNSCC [45] and has been associated with an increase in tumor cell motility and survival signaling [46–48].

Although the inhibition of cell proliferation in cells displaying high SERPINE1 expression may at first glance seem inconsistent with its oncogenic role, previous studies have pointed out that the enhanced migration induced by SERPINE1 expression may also be accompanied by a decrease in cell proliferation [49, 50]. It is reasonable to assume that changes in cell morphology necessary for cell motility and migration (e.g. cytoskeleton reorganization) are incompatible with those required for cell proliferation and division [51–55]. In line with the results obtained in head and neck cancer cells, SERPINE1 has also been described as a key player in wound healing and tissue remodeling programs by inhibiting cell proliferation and promoting epithelial cell migration [49]. Regional or distant metastases are the most common cause of death in patients with HNSCC. However, little is known about the mechanisms underlying their development. Our results suggest that SERPINE1 expression could be up-regulated during tumor cell transformation and this could result in an increase in the capacity of tumor cells to migrate, generate metastasis and develop resistance to genotoxic therapy and this is likely to have a negative impact on tumor response and patient clinical outcome. In the future, SERPINE1 expression could be included, together with other molecular and clinical variables, in diagnostic and therapeutic algorithms to predict clinical outcome in HNSCC patients. SERPINE1 could help to improve patient stratification and to develop personalized therapeutic approaches, thereby improving patient quality of life and survival. Just as SERPINE1 expression is being used to guide the administration of adjuvant chemotherapy in patients with node-negative breast cancer, in HNSCC patients it could be useful to help decide whether to intensify or de-intensify treatment according to their risk of recurrence. SERPINE1 levels could also be used to define the group of patients who should have a close clinical follow-up in order to anticipate the development of metastatic recurrences. Despite the positive association between SERPINE1 expression and poor clinical outcome observed in three independent cohorts, larger multicenter studies and clinical trials are warranted, using one of the pre-established cut-offs and techniques here described, to replicate our findings. This could validate SERPINE1 as a new biomarker useful for making treatment decisions in head and neck carcinoma patients.

In summary, a high expression of SERPINE1 is a poor prognostic marker in head and neck squamous cell carcinoma patients that increases the risk of metastatic recurrences after therapy, possibly due to an increase in tumor cell migration and in resistance to cisplatin.

## MATERIALS AND METHODS

### Patient characteristics, tissue samples and clinical follow-up

This study was performed analyzing two independent cohorts of patients with pathologically confirmed HNSCC. A retrospective study (*n* = 80) was performed using formallin-fixed paraffin-embedded (FFPE) pre-treatment tumor biopsies from patients at advanced stage (III, IVa and IVb) treated between 1995 and 2003 at Hospital de la Santa Creu i Sant Pau (HSCSP), Barcelona.

A second prospective study (*n* = 190) was run using fresh tumor biopsies obtained from patients treated at HSCSP (*n* = 167) and at Hospital Moises Broggi (*n* = 23), Sant Joan Despí, Barcelona, from 2002 to 2012. Twenty-four fresh mucosa samples were obtained from HNSCC patients in areas without visible lesions. Fresh samples were frozen in RNAlater (Life Technologies Ltd, UK) and kept in liquid nitrogen until processing and until de RT-PCR analyses. Tumor samples with < 80% of tumor tissue were excluded from the prospective study. Sixty-nine FFPE biopsies from patients included in this cohort were used to confirm SERPINE1 by immunohistochemistry. The study was approved by the local Ethics Committee and the Institutional Review Board at HSCSP, and informed consent was obtained from each patient. The study was conducted in accordance with REMARK guidelines and the declaration of Helsinki (Supplementary files, table S1) [56]. The median follow-up time was 68 months in the retrospective study and 37 months in the prospective study.

Local recurrence-free survival (LRFS) was defined as time from treatment initiation to recurrence at the primary site. Progression free-survival (PFS) was the time elapsed between treatment initiation and tumor progression. Tumor progression was considered as an increase in tumor size of 25% or higher, or the appearance of new lesions (local or regional recurrences, and distant metastases). Cancer-specific survival (CSS) was defined as time from diagnosis to death from cancer.

HPV status, detected using the short PCR-fragment-10 (SPF-10) assay (Lab. Biomedical Products, Rijswik, the Nederland's), was available for oropharyngeal tumors treated at HSCSP. A single patient with a HPV positive tumor was included in the retrospective study. In the prospective study, thirty-five tumors were HPV negative, 9 HPV positive and HPV status was not available in 20 patients.

We also analyzed SERPINE1 mRNA levels in 520 primary tumor samples and 44 normal mucosas obtained from HNSCC patients included in The Cancer Genome Atlas (TCGA) database (https://tcga-data.nci.nih.gov/tcga/). To analyze SERPINE1 expression in this cohort we used level 3 RNASeqv2 normalized expression values. Survival data were available for 507 patients.

### Immunohistochemistry

5-μm tissue block sections were deparaffinized in xylol and rehydrated using decreasing ethanol concentrations (100%, 96%, 80%, 70%, and 50%). For antigenic retrieval, samples were immersed in target retrieval solution, pH 9 (Dako, USA) and autoclaved for 10 minutes at 121°C. Endogenous tissue peroxidase was inactivated by immersing the samples in a 3% H_2_O_2_ solution for 10 minutes Samples were incubated with SERPINE1 monoclonal antibody (clone 1D5; Abnova, Taiwan) at 1:200 dilution. The EnVision™ FLEX and FLEX+ Visualization System was used for primary antibody detection following standard procedures. Two head and neck surgical samples and SCC9 cells over-expressing SERPINE1 were used as positive controls whereas negative controls were processed substituting the primary antibody by non-immunized mouse serum (Supplementary files, Figure S1). Immunostained sections were quantified by two independent observers using a Olympus BX51 microscope.

The percentage of positive cells (0–100%) and the overall intensity of staining (1, no staining or weak; 2, moderate; and 3, strong) were established analyzing five randomly choosen microscopic fields at 100x magnification for each sample. SERPINE1 expression was only evaluated in tumor cells.

There was inter-observer agreement in 95% of the samples; the remaining slides were re-evaluated and consensus decisions were made. Images were acquired using an Olympus DP72 digital camera and processed with CellD Imaging 3.3 software (Olympus).

### RNA purification and RT-PCR

RNA was isolated using Trizol reagent (Life Technologies Ltd, Paisley, UK) as previously described [57]. cDNA synthesis was performed using 1.5 μg of total RNA, 5 μL of RT buffer, 2 μL of dNTPs mixture, 5 μL of Random Hexamer Primers, 125 U of MultiScribe Reverse Transcriptase and 40 U of RNase inhibitor (Life Technologies Ltd, Paisley, UK), in a 50 μL final reaction volume with the High Capacity cDNA Archive Kit (Life Technologies Ltd, UK). Reaction conditions were 25°C for 20 minutes, 37°C for 2 hours and 95C for 3 minutes. Real Time RT-PCR reactions were performed in duplicate using the Hs01126607_m1 gene expression assay (Life Technologies Ltd, UK). HPRT1 amplification (Hs99999909_m1) was used as an endogenous control and RNA obtained from the UM-SCC-22A cell line was used as the calibration sample. Gene expression levels were expressed as fold change relative to the calibration sample (UM-SCC-22A), applying the comparative CT method (2^#x2212;ΔΔCT^). For UM-SCC-22A, RNA extraction and cDNA synthesis were performed following the same steps as described for tumor samples.

### Cell culture

SERPINE1 expression and proliferation, migration and apoptosis assays were performed using six human head and neck squamous cell carcinoma cell lines (UM-SCC-22A, UM-SCC-22B, UM-SCC-74B, SCC9, SCC25 and FaDu). 293T cells were only used to generate lentivirus-containing supernatants.

UM-SCC-22A, UM-SCC-22B, UM-SCC-74B [58] and 293T (ATCC^®^ CRL-3216™; ATCC;http://www.lgcstandards-atcc.org) cell lines were grown in Dulbecco's modified Eagle's medium (DMEM) containing 10% FBS, 100 U/mL streptomycin / penicillin and 2 mM glutamine (Life Technologies Ltd, UK). SCC-9 (ATCC^®^ CRL-1629™) and SCC-25 (ATCC^®^ CRL-1628™) HNSCC cell lines were grown in DMEM/F12 (1:1) containing 10% FBS, 100 U/mL streptomycin/penicillin, 2 mM glutamine and 0.4 μg/mL of hydrocortisone. FaDu (ATCC^®^ HTB-43™) HNSCC cell line from ATCC was grown in Dulbecco's modified Eagle's medium (DMEM) containing 10% FBS, 100 U/mL streptomycin/penicillin and 2 mM glutamine. All cell lines were cultured in a humidified atmosphere at 37°C and 5% of CO_2_. Cell lines were authenticated comparing the STR profiles obtained using the Cell ID kit (Promega Corporation, Madison, WI) with the original STR profiles previously described (Supplementary files, table S2) [58, 59].

### Generation of SERPINE1 over-expressing cell lines

cDNA-encoding human SERPINE1 was obtained from a pcDNA3.1-SERPINE1 plasmid (generously gift from Paul J. Higgins) and subcloned into the XhoI-BamHI site of the lentiviral vector FUtdTW obtained from Addgene (http://www.addgene.org/) [60, 61]. Lentiviral packaging was achieved after cotransfection of the vector plasmid with pMD.G_VSV G-poly-A vector and p8 91-Gag-Pol vector into 293T cells using lipofectamine 2000 kit (Life Technologies Ltd, UK). The lentivirus-containing supernatant was harvested 48 hours after 293T transfection, filtered through a 45 μm filter (Millipore) and stored at −80°C. UM-SCC-74B cells were transduced with the SERPINE1-expressing lentiviral vector (pFUGW_SERPINE1) and stable SERPINE1 over-expressing cells were selected exposing cells to 500 μg/mL zeocin for four weeks.

### Generation of SERPINE1 knockdown cell lines

Lentiviral vectors (pLKO.1-puro) containing short hairpin RNA (shRNA) against human SERPINE1 (TRC2_331004, TRC2_370159) and non-mammalian shRNA control plasmid DNA (scramble) were purchased from Sigma-Aldrich (Sigma Aldrich, MO, USA). Lentiviral packaging and transduction of SCC9 cells were performed as described for SERPINE1 over-expression. Stable SERPINE1 knockdown cells were selected in 10 μg/mL puromycin (Life Technologies Ltd, Paisley, UK). After four weeks of antibiotic selection, SERPINE1 expression was determined by Real Time PCR and Western blot.

### Proliferation, migration, cytotoxicity and apoptosis assays

Proliferation assays were performed seeding cells in the range of 3.5 × 10^4^ cells/well (UM-SCC-74B and vector-transduced cells) to 7 × 10^4^ cells/well (SCC9 and shRNA-transduced cells) in six-well plates. Cells were harvested after 24 h, 48 h, 72 h and 96 h of growth and counted using a Countess^®^ automated cell counter (Life Technologies Ltd, UK).

For the migration assays, cells were pre-incubated for 24 hours in FBS-free medium and seeded onto the upper chamber of a transwell cell culture insert diameter 6.5 mm, pore size 8 μm) (Corning, USA) in the presence of FBS-free medium. Each insert was introduced into a well (12-well plate) containing 500 μL of medium with FBS. After 24 hours incubation, cells at the top of the upper transwell chamber were removed by a mechanical action with gauze swabs and the external cells at the bottom of the transwell chamber were fixed for 10 minutes in methanol, stained with crystal violet for 10 minutes and dried at 37°C. Images were captured using the DP73 Olympus digital camera (Olympus Corporation, Japan). The extent of migration was determined by the area fraction occupied by cells in five ×100 fields using CellSens dimension v1.9 software (Olympus Corporation, Japan). Experiments were performed in triplicate and repeated three times.

For cytotoxicity assays, cells seeded in 96 well/plates (2500 cells/well), were exposed to cisplatin at a concentration ranging from 2.5 to 80 μM for 48 hours. We determined drug sensitivity measuring cell metabolic capacity using the XTT Cell proliferation kit II, as previously described (Roche Diagnostics, Germany) [62]. The half maximal inhibitory concentration (IC50) was calculated by linear interpolation as previously described [62].

To determine cisplatin-induced apoptosis, we treated cells with 15–40 μM cisplatin for 16 hours. After treatment, cells were fixed for 1 minute in methanol at −20°C and stained with ProLong^®^Gold Antifade DAPI (Life Technologies Ltd, Paisley, UK). Apoptotic and non-apoptotic nuclei were counted under a fluorescence microscope. We determined the mean percentage of apoptotic bodies assessing six 200X images per sample. MK-2206 (Selleckchem, Houston, TX, USA) and PAI-039 (Axon Medchem BV, Netherlands) inhibitors were used to study the AKT pathway activation.

### Western blot

Cell protein extracts and western blot analysis were performed as previously described [63]. Briefly, 75 μg of cellular protein extract was electrophoretically-separated and transferred to nitrocellulose membranes over-night. Membranes were blocked in TBS-T buffer [0.132 m NaCl, 0.02 m Tris (pH7.5), 0.1% Tween 20] containing 5 g/100 ml of milk for 1.5 hours and incubated over-night with primary antibodies. Dilutions for the primary antibodies were: SERPINE1(1:1000, MAB10390, Abnova, USA), Phospho-Akt (Thr308)(1:1000, #9275, Cell Signalling), Akt (1:500, Mouse anti-Akt clone 55, BD Biosciences), ERK(1:2500, clone 16/ERK, BD Biosciences, USA), PI3K (1:2500, clone 4/PI3-Kinase, BD Biosciences) and GAPDH (1:10000, MAB374, Merck Millipore, Germany).

### Statistical analysis

In the RT-PCR analysis and the TCGA database, the cut off to distinguish patients with high SERPINE1 expression and patients with low SERPINE1 expression was determined using Classification and Regression Tree Analysis (CART) [64]. CART analysis selected the cut-off with the best sensitivity and specificity to distinguish patients with a high risk of disease relapse from patients with low risk according to PA-1 expression.

Mann-Whitney and Kruskal-Wallis tests were used to assess the association between sex, age, tumor site, tumor size, node status, tumor differentiation, tobacco and alcohol consumption, rate of recurrences, with SERPINE1 expression. We distinguished between local (same anatomic site for recurrence and primary tumor) and metastatic (node recurrences or distant metastasis) recurrences. Kaplan-Meier analysis and Log-Rank test were used to analyze differences in LRFS, PFS and CSS between the subgroups of patients established according to SERPINE1 expression. A multivariate Cox model was used to test the association between sex, tumor size, node involvement, age or SERPINE1 mRNA expression with PFS and CSS. To analyze differences between two or more conditions in “*in vitro*” assays, we used the non-parametric Mann–Whitney U or Kruskal–Wallis tests.

Statistical analyses were performed using the SPSS v.22 (IBM Corporation, Armonk, NY) software. Differences were considered significant at *p*-values < 0.05 in all the applied tests.

## SUPPLEMENTARY FIGURES AND TABLES


