# Supplementary material for: Enhanced cell migration and apoptosis resistance may underlie the association between high SERPINE1 expression and poor outcome in head and neck carcinoma patients
Source: Oncotarget. 2015 Aug 24;6(30):29016–33. doi: 10.18632/oncotarget.5032 (PMC4745708; doi:10.18632/oncotarget.5032)
Supplement: Supplementary file 1 [file oncotarget-06-29016-s001.pdf]

SUPPLEMENTARY FIGURES AND TABLES

Supplementary Table S1: Report of compliance of REMARK criteria*

**Introduction**<br>SERPINE1 (PAI-1) was selected as the candidate marker for this study. We studied the prognostic value of SERPINE1 (PAI-1) expression in head and neck squamous cell carcinoma (HNSCC) in a retrospective and a prospective studies in independent patient cohorts. Hypothesis and objectives are stated in introduction section ***(Pages 3–4)***
**Materials and Methods**<br>**Patients**<br>Patients with pathologically confirmed HNSCC were eligible for the study. The inclusion in the study did not modify patient treatment. Patients received a standard treatment according to their stage. Patient characteristics and treatment are described in table 1 and table 2. Inclusion and exclusion criteria are described in the methods section ***("Patient characteristics, tissue samples and clinical follow-up") (Pages 16–17)***
**Specimen characteristics**<br>The retrospective study was performed using paraffin-embedded pre-treatment tumor biopsies, whereas in the prospective study, we used pre-treatment fresh tumor biopsies. The collection, freezing and preservation of samples is detailed in the methods section ***("Patient characteristics, tissue samples and clinical follow-up") (Pages 16–17)***
**Assay methods**<br>SERPINE1 (PAI-1) expression was evaluated by Immunohistochemistry and real time RT-PCR. Details of sample processing and expression analysis are described in methods. Analysis were performed blinded to the study end point ***("Immunohistochemistry" & "RNA purification and RT-PCR") (Pages 18–19)***
**Study design**<br>Patients were recruited retrospectively and prospectively. A retrospective study (n = 80) was performed using pre-treatment tumor biopsies from patients treated between 1995 and 2003 at Hospital de la Santa Creu i Sant Pau (HSCSP), Barcelona. A prospective study (n = 190) was run using fresh tumor biopsies obtained from patients treated at HSCSP and at Hospital Moises Broggi from 2002 to 2012. The median follow-up time was 68 months in the retrospective study and 37 months in the prospective study. The primary end points analyzed were local recurrence-free survival (LRFS), progression-free survival (PFS) and cancer-specific survival (CSS). Their description and secondary end points are defined in methods ***("Patient characteristics, tissue samples and clinical follow-up") (Pages 16–17)***
**Statistical analysis methods**<br>Classification and regression tree analysis was used to define patient subgroups based on SERPINE1(PAI-1) mRNA expression and their risk of disease relapse. The association between SERPINE1 (PAI-1) expression and clinicopathological variables was assessed using Mann Whitney and Kruskal Wallis tests. Kaplan-Meier analysis and Log-Rank test were used to analyze the association between the tumor subclassification and time to event outcome. Univariate and multivariate Cox models were used to test the association between clinical variables and SERPINE1 (PAI-1) expression with PFS and CSS ***("Statistical analysis") (Pages 23–24).***
**Results.**<br>**Data**<br>The number of patients in each subgroup is detailed in the Kaplan Meier survival curves or tables. Basic demographic characteristics of the studied patients and standard prognostic variables are described in **table 1 and 2**.
**Analysis and presentation**<br>The association between SERPINE1 (PAI-1) expression and standard prognosis variables are detailed in Table 1 and 2. SERPINE1 (PAI-1) expression was associated with the appearance of local or metastatic recurrences after treatment. Kaplan-Meir and log rank test showed an association between SERPINE1 (PAI-1) expression and PFS and CSS (Figure 1–3). Multivariate analysis showed that PAI-1 expression, node stage and tumor size were associated with PFS (Table 3–4). Hazard ratios, confidence intervals and significance are detailed in tables, figures and described in results section **(Pages 5–11).**
**Discussion**<br>We showed that a high SERPINE1 (PAI-1) expression was associated with poor clinical outcome in patients with HNSCC. Larger multicenter studies and clinical trials are warranted to further validate the role of SERPINE1(PAI-1) in treatment decisions. The clinical implications and value of our findings are stated in the discussion section. **Pages 12–16**

* McShane LM, Altman DG, Sauerbrei W, Taube SE, Gion M, Clark GM. Reporting recommendations for tumor marker prognostic studies (REMARK). J Natl Cancer Inst 2005; 97(16):1180–4.

Supplementary Table S2: Authentication of the HNSCC cell lines used in the study

a) STR profiles for the HNSCC cell lines analyzed in the study obtained using the Cell ID system

CELL LINE	AMELOGENIN	CSF1PO	D13S317	D16S539	D5S818	D7S820	THO1	TPOX	vWA	D21S11
SCC-9	X,Y	11	9	10, 11	12	8	8,9	9,11	17	ND
SCC-25	X	10	13	11, 12	12	12	8	8,12	17,19	ND
FaDu	ND	12	8, 9	11	12	11, 12	8	11	15, 17*	31.2
UM-SCC-22A	X	10	8, 12	9, 11	12, 12	8,9	6	8, 11	15, 18	28
UM-SCC-22B	X	10	8, 12	9, 11	12	8, 9	6	8, 11	15, 18	28
UM-SCC-74B	X	12	12	10, 12	12	11	6, 9.3	8	15, 16	30, 34.2
K565 [1]	X	9, 10	8	11, 12	11, 12	9, 11	9.3	8,9	16	29, 30, 31

[1] Cell line used as an intrinsic control ND: Not detected

* allelic loss

Cell line authentication was performed using the Cell ID system (Promega, USA) following manufacturer's instructions. DNA was extracted from cells using the Wizard Genomic DNA purification Kit (Promega, USA) and quantified spectrophotometrically. For each cell line, 200 ng of DNA was amplified in a GeneAmp PCR 9700 system. STR markers were analyzed using a 3130xl Genetic Analyzer and GeneMapper v4.0 software.

b) Reference STR profiles

CELL LINE	AMELOGENIN	CSF1PO[4]	D13S317	D16S539[4]	D5S818	D7S820	THO1[4]	TPOX[4]	vWA	D21S11[5]
SCC-9 [1]	X,Y	11	9	10, 11	12	8	8,9	9,11	17	–
SCC-25 [1]	X	10	13	11, 12	12	12	8	8,12	17,19	–
FaDu [1]	ND	12	8, 9	11	12	11, 12	8	11	15, 17, 18	–
UM-SCC-22A[2]	X	–	8, 12	–	12, 12	8,9	–	–	15, 18	28
UM-SCC-22B[2]	X	–	8, 12	–	12	8, 9	–	–	15, 18	28
UM-SCC-74B[2]	X	–	12	–	12	11	–	–	15, 16	30, 34.2
K565 [3]	X	9, 10	8	11, 12	11, 12	9, 11	9.3	8,9	16	29, 30, 31

[1] Original STR profiles described in the ATCC Data Base (https://www.lgcstandards-atcc.org)

[2] Original STR profiles for the UM-SCC cell lines described by Brenner et al. (Brenner, JC. Head and Neck 2010)

[3] Original STR profile for the K565 cell line used as an intrinsic control

[4] CSF1PO, D16S539, THO1 and TPOX STR loci were not included in the Profiler Plus PCR Amplification Kit (Applied Biosystems, Foster City, CA) used in the study described by Brenner et al.

[5] D21S11 STR locus was not included in the ATCC Data Base.

ND: Not detected

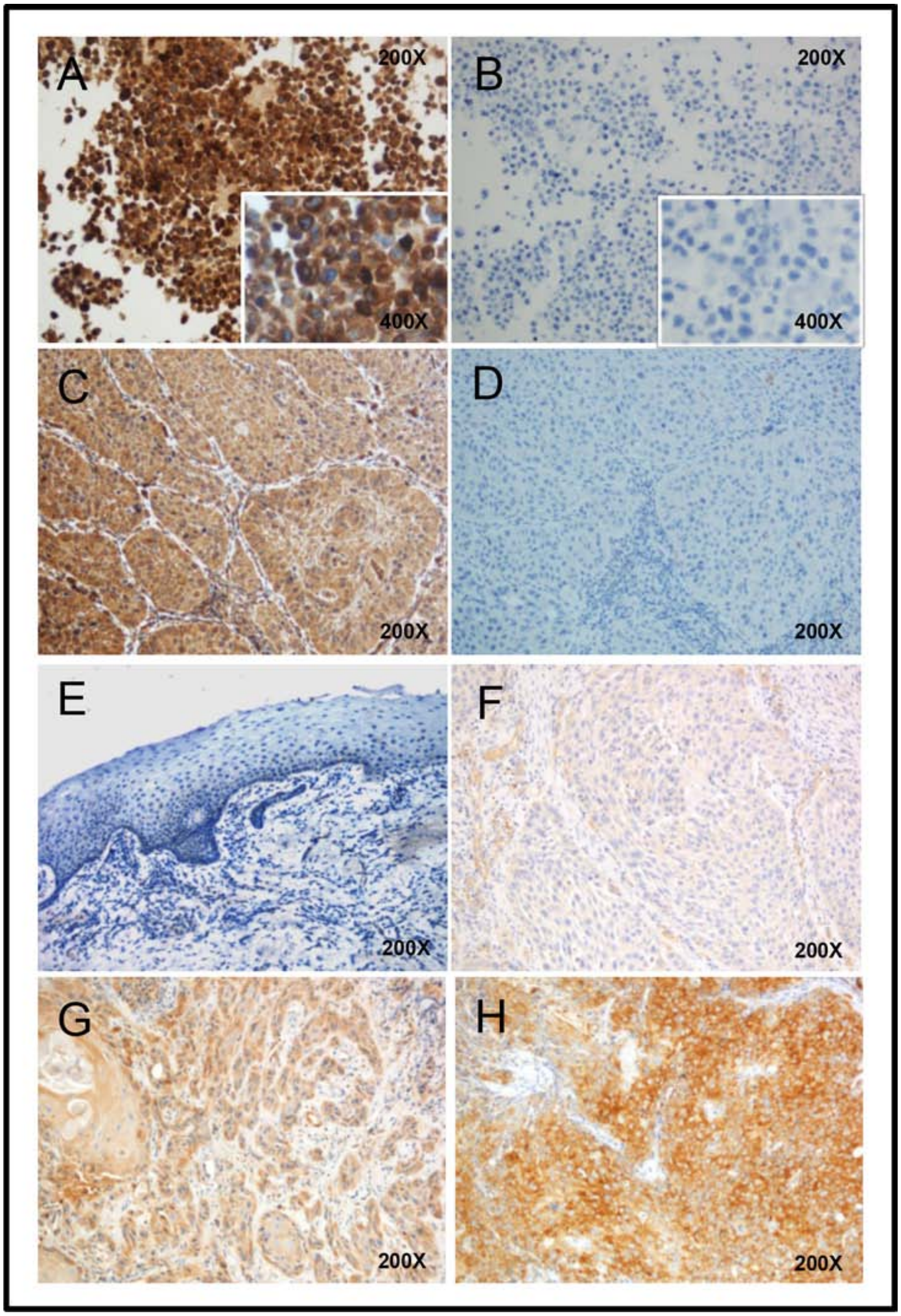

Supplementary Figure S1: Representative images of SERPINE1 immunohistochemistry in tumors with different levels of expression with the use of positive and negative tumor and normal tissue controls. Representative images of SERPINE1 immunohistochemistry **A, C.** Positive controls (SCC9 cells and surgical sample of HNSCC tumor); **B, D.** Negative controls (SCC9 cells and surgical sample of HNSCC tumor without a primary antibody incubation); **E.** normal mucosa adjacent to tumor tissue; **F.** Tumor tissue with low SERPINE1 expression (+), **G.** intermediate SERPINE1 expression (++) and **H.** high SERPINE1 expression (+++).

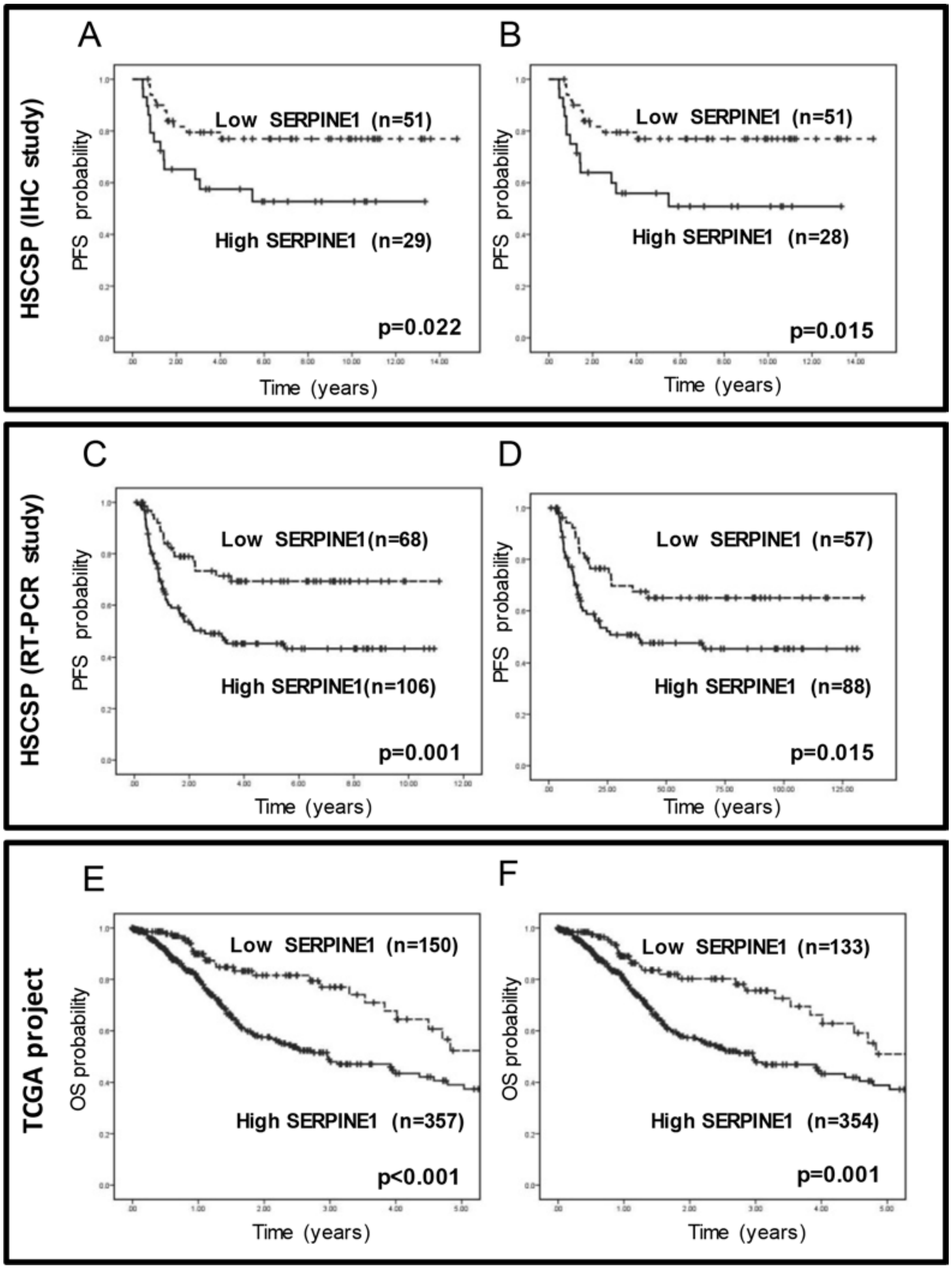

Supplementary Figure S2: Poor prognosis of SERPINE1 overexpression after the exclusion of the HPV positive tumors. Patients bearing tumors with high SERPINE1 expression continue to have poorer survival in the three analyzed cohorts (left panels: **A, C & E.**), after excluding HPV positive patients from each cohort (right panels: **B, D & F.**). Differences in Progression-free survival (PFS) between high ($n = 28$) and low ($n = 51$) SERPINE1 expressing groups were maintained in the retrospective HSCSP IHC study (B, $p = 0.015$); Differences in Progression-free survival (PFS) between high ($n = 103$) and low ($n = 62$) SERPINE1 expressing groups were maintained in the HSCSP-RT-PCR based study after excluding patients with oropharyngeal HPV positive tumors or oropharyngeal tumors which HPV status was unknown (D, $p = 0.015$); Differences in Overall survival (OS) between high ($n = 354$) and low ($n = 133$) SERPINE1 expressing groups were maintained in the TCGA-based study (F, $p = 0.001$).
